# Stromal Fibrosis of the Breast: A Spectrum of Benign to Malignant Imaging Appearances

**DOI:** 10.1155/2019/5045908

**Published:** 2019-02-03

**Authors:** Lara Nassar, Amro Baassiri, Fatima Salah, Andrew Barakat, Elie Najem, Fouad Boulos, Ghina Berjawi

**Affiliations:** ^1^Department of Diagnostic Radiology, American University of Beirut Medical Center, Lebanon; ^2^Department of Pathology, American University of Beirut Medical Center, Lebanon

## Abstract

**Objectives:**

The aim of this study is to demonstrate the various imaging appearances of stromal fibrosis on mammography, ultrasound, and magnetic resonance imaging (MRI).

**Material and Methods:**

This study included 75 female patients who presented to the American University of Beirut Medical Center between January 2010 and October 2015 for breast imaging. 66 (88%) patients obtained a mammogram, 71 (95%) had an ultrasound, and 6 (8%) had an MRI. Patients included had stromal fibrosis proven on biopsy.

**Results:**

The most common finding on mammogram was calcifications which was present in 14 (21%) patients, while on ultrasound it was a mass which was present in 61 (86%) patients. A mass was detected on MRI in 2 (33.5%) patients. Most lesions detected had benign findings such as masses with circumscribed margins. We had a follow-up for 53 (71%) patients with an average follow-up interval of 28.5 months (range: 5 – 70). Increase in size of the index lesion was noted in only 2 patients; upon rebiopsy, pathology results read stromal fibrosis for one lesion and fibroadenoma for the other. The remaining lesions were either stable or decreased in size. The higher detection rate of a mass on ultrasound was statistically significant (p<0.001) in comparison to that of mammography.

**Conclusion:**

Stromal fibrosis can have various presentations on imaging from benign to suspicious for malignancy features. In the case of accurate targeted biopsy, when stromal fibrosis is diagnosed, the result can be considered concordant. Therefore, such lesions can be followed up by imaging to document stability and confirm benignity.

## 1. Introduction

Stromal fibrosis in the breast is a benign pathologic entity. It is primarily characterized by proliferation of fibrous tissue that results in the obliteration of the mammary acini and ducts [1]. The exact etiology remains unknown. However, it has been speculated that it may be related to several conditions such as estrogen related fibroblastic proliferation, end stage of inflammatory processes, or a type of breast involution [2]. In this era of screening mammography, it has become a common diagnosis in percutaneous image-guided breast biopsy. Pathologists have described this entity by various terms which include “focal fibrosis” of the breast and “fibrous mastopathy” [3]. Collectively, it has been reported to represent as high as 9% of the lesions biopsied [4].

Stromal fibrosis may present as a palpable mass or a clinically occult imaging-detected abnormality [4]. On imaging, it may present with either benign or suspicious features. The major dilemma lies in the case of radiologic-pathologic discordance, for it is one of the leading causes of missed diagnosis of breast cancer [5]. Discordance may result in a second core biopsy or even a more invasive procedure such as prompting a surgical excision of the lesion in order to avoid misdiagnosing malignant lesions [6]. Literature is almost scarce regarding the eclectic radiological features this entity may present with. It is vital for radiologists to recognize that this benign entity may present as a suspicious lesion on imaging, for it will make the assessment of concordance easier. Therefore, the aim of this study is to demonstrate the various imaging appearances of stromal fibrosis on mammography, ultrasound, and magnetic resonance imaging (MRI).

## 2. Materials and Methods

### 2.1. Patient Selection

This institutional review board (IRB) approved retrospective study included 75 female patients who presented to the American University of Beirut Medical Center (AUBMC) between January 2010 and October 2015 in which pathology examination detected only “stromal fibrosis”, “focal fibrosis”, or “fibrous mastopathy”. Those who had multiple biopsies in the same breast at presentation where cancer was diagnosed in any of the sites biopsied were excluded. However, patients diagnosed with a contralateral breast cancer were not excluded.

### 2.2. Image Interpretation

A fellowship trained board certified radiologist with 5 years of experience in breast imaging and a senior resident in radiology interpreted the images by consensus. The mammograms were assessed for breast density (extremely dense, heterogeneously dense, scattered fibroglandular, and almost entirely fat), masses (shape, margin and size), calcifications (morphology and distribution), and architectural distortion. Ultrasound images were assessed for masses (shape, margin, size, echo pattern, and posterior features if any), calcifications, and special features such as intracystic mass. MRI images were assessed for masses (shape, margin, size, and internal enhancement characteristics if any), nonmass enhancements (distribution and internal enhancement characteristics if any), and kinetic curve. Changes in the lesion on follow-up images were noted. The American College of Radiology BI-RADS Atlas Fifth Edition was used as reference for image evaluation [7].

### 2.3. Chart Review

Patient charts were retrieved from the medical records and were reviewed for the following data: patient age, indication for presentation (screening versus diagnostic), chief complaint on presentation, family history of breast cancer, follow-up period, and pathology reports.

### 2.4. Statistical Analysis

Statistical analysis was performed on the SPSS software (release 23.0; SPSS Inc.) for Windows (Microsoft). Quantitative data were described as mean, range (minimum-maximum). Qualitative parameters were compared using the Chi-Square test. Results were considered statistically significant at p≤0.05 (2-sided).

## 3. Results

We had a total of 75 patients with a mean age of 47 years (range: 20–76 years). 33 women were presenting for their yearly screening and 42 for diagnostic purposes (palpable breast/axillary mass, bloody nipple discharge, and breast pain). 66 (88%) patients obtained a mammogram, 71 (95%) patients obtained an ultrasound, and 6 (8%) patients had a MRI. Four (5.5%) patients had all 3 imaging modalities done, 62 (82.5%) patients had a mammogram and an ultrasound, and 2 (2.5%) patients had only a mammogram and an MRI. Mammography was performed as the initial examination in women above age of 40 presenting for screening or diagnostic purposes, followed by ultrasound for evaluation of suspicious findings or for screening in dense breasts. Ultrasound was the initial exam in women younger than 40, followed by mammography if suspicious findings were present. MRI was performed in 4 high risk patients (prior personal history of breast cancer, prior radiation to the chest, or positive family history for breast cancer), in 1 patient upon her specific request and was the initial test performed in another facility in one woman presenting to our institution for a biopsy.

### 3.1. Mammography Findings

Mammography findings are summarized in Table 1. The frequencies of the 4 breast densities were as follows: extremely dense, 47 (71%); heterogeneously dense, 7 (10.5%); scattered fibroglandular, 7 (10.5%); almost entirely fat, 5 (7.5%).

**Figure 1:**
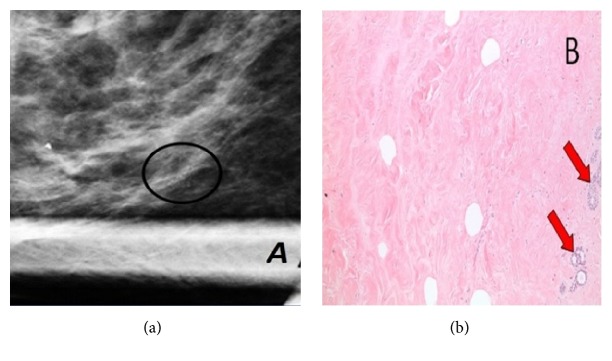
57-year-old woman presenting for screening mammography. (a) Spot magnification view of the right breast in mediolateral position shows segmental amorphous calcifications. A follow-up mammogram was obtained 7 months following the biopsy showing stable appearance. (b) H&E stain of the biopsied tissue shows dense collagenous stroma with rare entrapped fat cells and involuted entrapped ducts (arrows).

**Figure 2:**
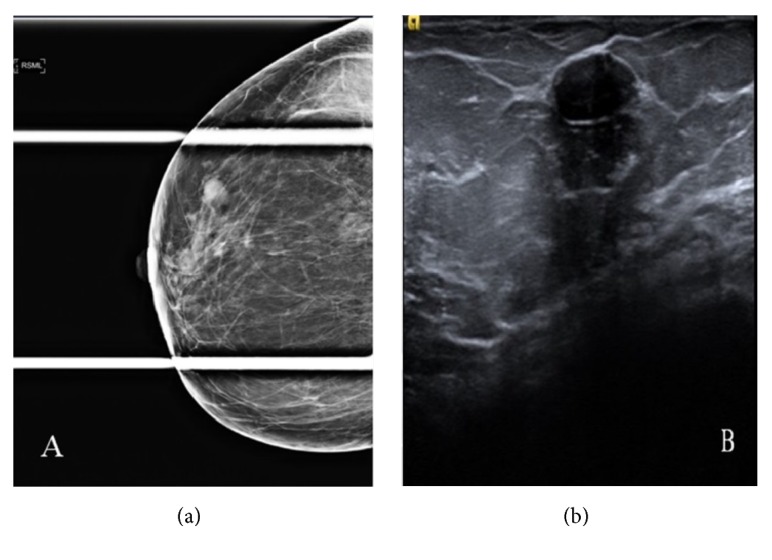
53-year-old woman presenting for evaluation of a palpable mass. (a) Spot compression view at the site of the palpable abnormality shows an oval circumscribed equal density mass. (b) Ultrasound confirms an oval circumscribed hypoechoic mass with parallel orientation. A biopsy was recommended in view of the new palpable finding. The mass remained stable at follow-up after 18 months.

**Figure 3:**
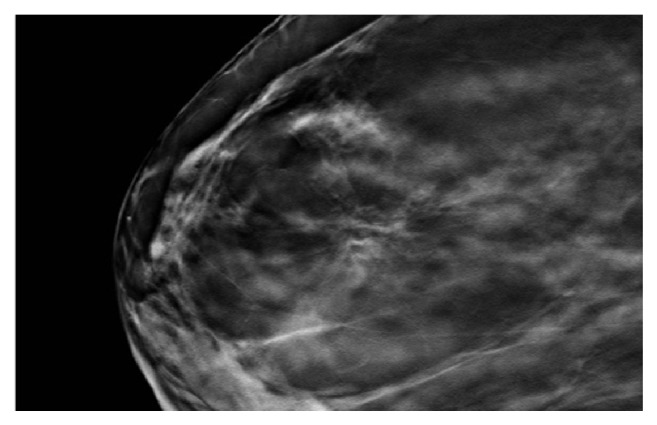
72-year-old woman presenting for a screening mammography. Cropped image from a tomosynthesis view of the right breast in mediolateral oblique position showing an area of architectural distortion. The biopsy revealed stromal fibrosis. Surgical excision was performed and the final surgical pathology also confirmed stromal fibrosis.

The most common finding on mammogram was calcifications which was present in 14 (21%) patients. Amorphous calcifications were demonstrated in 9 (64%) patients. The rest of the morphologies were seen each in a single patient: punctate, pleomorphic, coarse heterogeneous, round, and linear. Concerning their distribution, calcifications were grouped in 10 (71.5%) patients, segmental in 2 (14.5%) patients (Figure 1(a)), diffuse in one patient, and regional in another. Furthermore, a mass was seen in 11 (17%) patients. The smallest mass detected had the dimensions of 8x5mm, while the largest mass was 40x44x25mm. The most common shape was an oval (Figure 2(a)), while it was irregular in one patient and round in another. Most of the masses detected were circumscribed; however, the margin of the mass was obscured in 3 (27%) patients and indistinct in 2 (18%) patients. Architectural distortion (Figure 3) was detected in 2 (3%) patients. The mammogram was negative in 39 patients.

### 3.2. Ultrasound Findings

**Figure 4:**
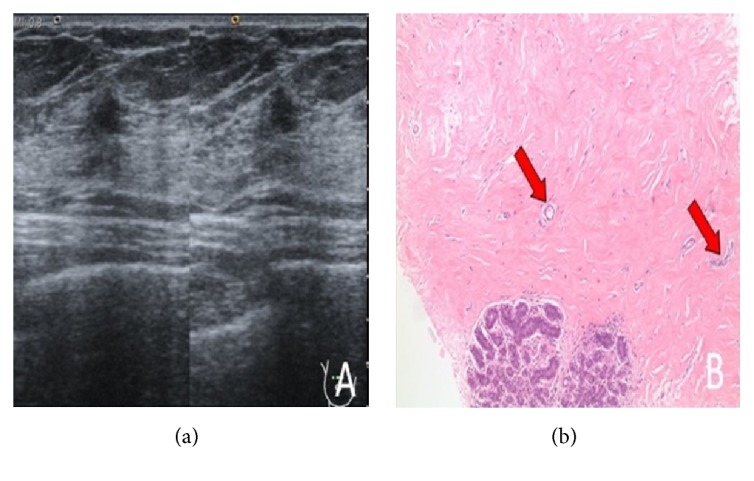
41-year-old woman presenting for screening. Mammography (not shown) demonstrated heterogeneously dense breast with no suspicious abnormality. (a) Screening ultrasound shows a hypoechoic mass with irregular shape and margin. The patient was lost to follow-up after the biopsy. (b) Dense fibrous stroma with small capillaries (arrows) and adjacent appearing lobular units.

**Figure 5:**
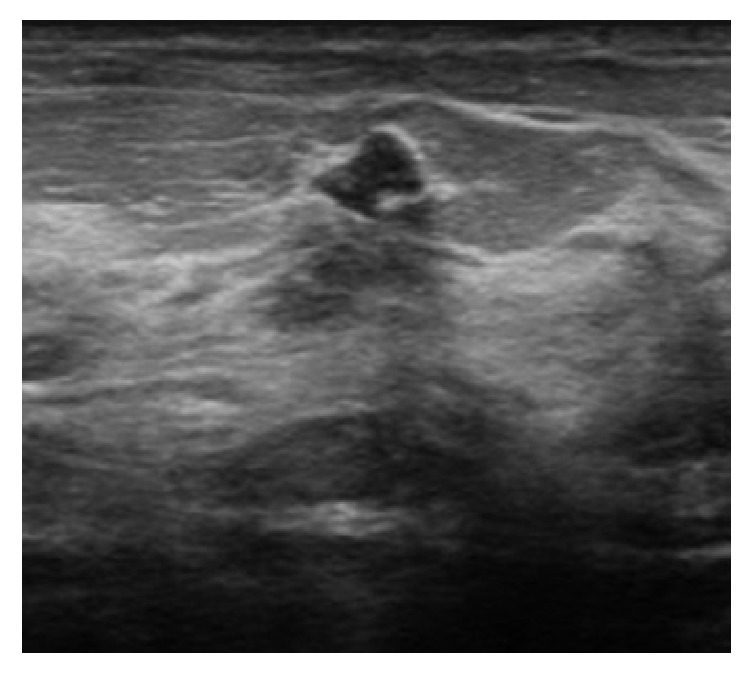
40-year-old woman presenting for screening mammography and ultrasound. Mammography (not shown) revealed dense breasts and no focal lesions. Ultrasound showed a hypoechoic mass with angular margins. Biopsy shows stromal fibrosis. Follow-up after two years showed no malignancy.

The most common finding on ultrasound was a mass which was present in 61 (86%) patients (characteristics summarized in Table 2). The smallest mass detected was 3mm, while the largest mass had the dimensions of 41x44x21mm. The most common shape was oval which was seen in 33 (54%) patients. An irregular mass was seen in 24 (39%) patients (Figure 4(a)), while a round mass was seen in 4 (6.5%) patients. Most of the masses detected were circumscribed which was detected in 18 (29.5%) patients. Indistinct and irregular margins were detected in an equal number of 15 (24.5%) patients. Microlobulated margins were detected in 9 (15%) patients, while angular margins (Figure 5) were detected in 4 (6.5%) patients only. In addition, we had various echo patterns; a hypoechoic mass was the most common (Figure 2(b)). Other patterns observed were as follows: heterogeneous, isoechoic, and complex cystic-solid masses. Moreover, 2 masses were intracystic. Most masses did not have a posterior acoustic feature. However, posterior shadowing was seen in 15 (24.5%) patients and posterior enhancement was seen in 2 (3.5%) patients. Furthermore, calcifications were seen in 5 (8%) patients. The remainder of the patients had no ultrasound findings.

### 3.3. MRI Findings

**Figure 6:**
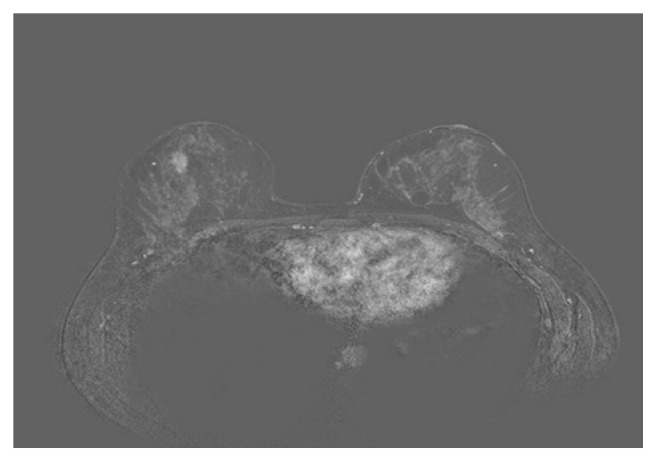
33-year-old high risk woman (prior history of irradiation to the chest). Screening MRI shows a focal nonmass enhancement in the right breast. Biopsy shows stromal fibrosis. Initial follow-up MRI after 6 months shows stable appearance. Further screening MRI scans show slight regression in size.

MRI findings are summarized in Table 3. A mass was detected on MRI in 2 (33.5%) patients with dimensions of 7x4mm and 11x7mm. One mass was oval while the other was irregular in shape. Both mass margins were irregular. One mass had an enhancing rim while the other was homogenous. The last patient with a prior history of contralateral breast cancer had negative MRI finding. Clumped segmental nonmass enhancement (Figure 6) was detected in 3 (50%) patients. Three patients had kinetic curve I. Kinetic curves II and III were seen in one patient each.

### 3.4. Pathology Results and Follow-Up

The pathology reports read stromal fibrosis (Figures 1(b) and 4(b)) for all the patients except for one which read fibrous mastopathy. The selected cases show a similar array of histopathologic findings. These include dense stromal fibrosis (as opposed to the normal loose collagenous mammary stroma found in unaffected breast parenchyma), paucity of lobular units within the fibrous areas, and occasionally entrapped fat cells. The fibrous areas also characteristically include small capillary-sized vessels. Epithelioid fibroblasts such as the ones present in diabetic mastopathy were not seen in these cases. Also, the fibrous areas tended to interface with normal appearing breast stroma in some of the tissue-rich core biopsies. We had a follow-up for 53 (71%) patients with an average follow-up interval of 28.5 months (range: 5 – 70). 46 patients (87%) had a stable lesion with no interval history of malignancy. Four (7.5%) patients had a decrease in the size of the lesion, while 2 (4%) other patients had an increase in size. Finally, we had one patient who had a total ipsilateral mastectomy in another hospital before presenting back to us 5 months later. The indication that led the patient to undergo a surgery and the resulting pathology are unknown. As for the patients who had an increase in lesion size, their lesions were biopsied once again. The pathology report read fibroadenoma for one patient and stromal fibrosis for the other.

On chi-square test, the higher detection rate of a mass on ultrasound was statistically significant (p<0.001) in comparison to that of mammography. There was no significant difference in detecting calcifications (p=1.000) between the same imaging modalities.

## 4. Discussion

The pathogenesis of stromal fibrosis remains unknown. However, the theory of a hormonal role has gained support by previous studies whereby patients were almost exclusively premenopausal [3, 8]. However, similar to Taskin et al. [9] who reported a high percentage of 33% of postmenopausal women with stromal fibrosis, 25% of patients in our study were postmenopausal. This does not rule out a possible hormonal role; nevertheless, other explanations ought to be pursued. Furthermore, only 16% of the lesions detected were palpable. This is resonated in other studies where nonpalpable lesions make up 65%-100% of the cases [1, 10, 11]. This may be primarily due to increased utilization of breast ultrasound and MRI for breast cancer screening whereby lesions are being detected before they increase in size and become palpable. 

The imaging features of stromal fibrosis are not specific and variable. On mammography, the most common finding in our study was calcifications followed by that of a mass although this did not reach statistical significance, unlike other studies which report mass lesion as the most common mammographic abnormality [4, 6, 9]. In fact, Harvey et al. [3] had a sample of 15 lesions where none of the lesions had calcifications. Most lesions we encountered had probably benign findings such as masses with circumscribed margins. Few lesions had findings more worrisome for malignancy such as architectural distortion, irregular masses with indistinct margins, and pleomorphic calcifications with a segmental distribution. Other studies have documented suspicious findings such as spiculated masses more frequently within their sample [9]. On ultrasound, though a well-defined mass was the most common finding, a big proportion of lesions presented as irregular masses with irregular, microlobulated, or angular margins. The masses presented with various echo patterns, namely, hypoechoic, heterogeneous, isoechoic, and complex cystic-solid. Although most masses had no posterior acoustic features, 24.5% of the lesions had posterior shadowing. Similar ultrasound findings were reported in other studies [1, 4, 6, 9]. A higher detection rate of mass lesions on ultrasound was found to be statistically significant when compared to mammography. This could primarily be due to the fact that 81.5% of our patients had dense breasts which could limit mammography.

All lesions detected on MRI had findings suspicious for malignancy. Three lesions presented with clumped segmental nonmass enhancement. Two other mass lesions had irregular margins. One of those lesions was an irregular mass with rim enhancement. Lee et al. [11] had a larger sample of cases that had undergone MRI where most lesions presented as masses unlike our findings where nonmass enhancements were most common. However, similarly to our study, most nonmass lesions presented with clumped enhancement.

Our study has the second longest follow-up for stromal fibrosis lesions in the literature to date with a mean of 28.5 months (range: 5–70). No false negatives are to be reported. None of the patients had an interval history of malignancy. On follow-up, only 2 patients had an increase in lesion size which upon rebiopsy had pathology results that read stromal fibrosis for one and fibroadenoma for the other. Jai Kyung et al. have also demonstrated lesion size stability and/ or decrease over a 2-year follow-up period with no false negatives [12]. Similarly, no false negatives were noted by Rosen et al. [4] and Harvey et al. [3] who reported rebiopsy of lesions increasing in size, proven again to be benign (focal fibrosis and fibroadenoma respectively). No false negatives were reported by Lee et al. [11] in his series of 40 cases of stromal fibrosis detected on MRI. The false negatives reported in the literature are related to either sampling error as noted by Sklair-Levy et al. [1] who describe two cases of invasive carcinoma mistakenly labeled as stromal fibrosis and to radiological-pathological discordance according to Malik et al. [13] who reported a false negative rate of 7%.

A limitation of this study is the relatively small sample size that resulted from the inclusion criteria. In addition, we had lost 22 patients to follow-up.

## 5. Conclusion

Although stromal fibrosis is most likely to occur as an ultrasound detected mass, this study has not identified a characteristic imaging appearance. In fact, stromal fibrosis may demonstrate a wide array of radiological phenotypes on mammography, ultrasound, and MRI, displaying features ranging from benign to suspicious for malignancy. In the case of accurate targeted biopsy, when stromal fibrosis is diagnosed, the result can be safely considered concordant. It is currently recommended that these patients undergo a follow-up ultrasound for documentation of benignity. Our study parallels several others that show no false negative results upon follow-up. In light of this reproducible finding across several studies, larger studies are needed in order to reconsider the need for short-term follow-up ultrasound after diagnosis of stromal fibrosis.

## Figures and Tables

**Table 1:** Mammographic findings.

Calcification Characteristics	Number of lesions	Mass Characteristics	Number of lesions
*Morphology*		*Shape*	
Amorphous	9	Oval	9
Punctate	1	Round	1
Pleomorphic	1	Irregular	1
Coarse heterogeneous	1		
Round	1		
Linear	1		

*Distribution*		*Margin*	
Grouped	10	Circumscribed	6
Segmental	2	Obscured	3
Diffuse	1	Indistinct	2
Regional	1		

*Total*	*14*	*Total*	*11*

**Table 2:** Mass characteristics on ultrasound.

Mass Characteristics	Number of lesions
*Shape*	
Oval	33
Round	4
Irregular	24

*Margin*	
Circumscribed	18
Indistinct	15
Irregular	15
Microlobulated	9
Angular	4

*Echo Pattern*	
Hypoechoic	54
Heterogeneous	4
Complex cystic	2
Isoechoic	1

*Posterior Features*	
Shadowing	15
Enhancement	2
None	44

*Total*	*61*

**Table 3:** MRI findings.

Mass Characteristics	Number of lesions	Non-Mass Characteristics	Number of lesions
*Shape*		*Distribution*	
Oval	1	Segmental	3
Irregular	1		

*Margin*		*Internal Enhancement Pattern*	
Irregular	2	Clumped	3

*Internal enhancement*			
Rim enhancement	1		
Homogeneous	1		

*Total*	*2*	*Total*	*3*

## Data Availability

The data used to support the findings of this study are available from the corresponding author upon request.
